# Fabrication of a Novel PES/CNTs@TiO_2_ Membrane Combining Photo-Electrocatalysis and Filtration for Organic Pollutant Removal

**DOI:** 10.3390/membranes15030090

**Published:** 2025-03-10

**Authors:** Yue Lu, Xuan Xing, Yi Jiang, Jianxin Xia

**Affiliations:** 1 College of Life and Environmental Science, Minzu University of China, Beijing 100081, China; luyue0122@126.com; 2 College of Chemistry and Environmental Engineering, Hohhot Minzu College, Hohhot 010051, China; 3 Department of Civil and Environmental Engineering, The Hong Kong Polytechnic University, Hung Hom, Kowloon, Hong Kong; yi-cee.jiang@polyu.edu.hk

**Keywords:** membrane, CNTs@TiO_2_, photo-electrocatalysis, flux, service life

## Abstract

Membrane filtration has been widely used in wastewater treatment; contaminants attached to the membrane surface led to flux loss and service life reduction. In the present study, a photo-electrocatalysis membrane was fabricated with CNTs@TiO_2_ deposited on a commercial polyethersulfone (PES) membrane (PES/CNTs@TiO_2_). XRD and SEM characterization proved that the CNTs@TiO_2_ composites were successfully fabricated using the one-pot hydrothermal method. Additionally, vacuum filtration was used to distribute the as-prepared powder on the PES membrane. In CNTs@TiO_2_, TiO_2_ particles were deposited on the outer layer of CNTs, which benefits light adsorption and photocatalytic reaction. The hydrophilicity, light absorption ability, and electron transfer rate of the PES/CNTs@TiO_2_ membrane were enhanced compared with the pristine PES membranes. Organic compound removal was improved in the photo-electrocatalysis filtration system with the improvement of 32.41% for methyl orange (MO), 26.24% for methyl blue (MB), 7.86% for sulfamethoxazole (SMZ), and 25.19% for florfenicol (FF), respectively. Moreover, the hydrophilicity and removal rate could be restored after pure water cleaning, demonstrating excellent reusability. The quenching experiment showed that ·OH and ·O_2_^−^ were the main reactive oxygen species. This work provides a convenient form of photo-electrocatalysis filtration technology using modified commercial membranes, which has great potential for practical application.

## 1. Introduction

In the course of industrial development, numerous organic pollutants, including phenol [1], medicines [2], and dyes [3] have been widely discharged into the environment, which poses a significant threat to human health [4]. Therefore, the development of efficient technology for contaminant removal is an urgent priority. Compared with the biological method, which is easily affected by contaminant toxicity, and advanced oxidation processes (AOPs), whose costs are high [5,6,7], membrane-based filtration technology has shown great potential in practical application due to its superior separation efficiency, low energy consumption, and environmentally sustainable features [8,9,10,11,12,13].

However, membrane fouling caused by organic contaminants during filtration is unavoidable, ultimately lowering the permeability and selectivity significantly [14,15,16]. This challenge results in high operational costs for large-scale plants due to frequent cleaning and premature module replacement [17]. Although conventional strategies like backwashing and chemical cleaning offer temporary relief, they are limited by mechanical wear, secondary pollution from cleaning agents, and only minor improvements in membrane stability [18]. To address these issues, combining membrane filtration with advanced oxidation processes (AOPs) has proven to be an effective solution [19,20,21,22,23].

Among all the AOPs, membrane separation combined with photocatalysis oxidation has attracted increasing attention due to its strong anti-fouling potential and disinfection capabilities [24]. This approach allows for simultaneous separation and oxidation of organic pollutants, enhancing membranes’ long-term stability and reusability. However, efficiency is limited by the rapid recombination of photo-generated electrons and holes. The photo-electrocatalytic (PEC) method addresses this limitation by using externally applied bias voltages to separate electron–hole pairs, facilitating the sustained generation of reactive oxygen species (ROS) for the mineralization of foulants [25]. As summarized in Table 1, integrated membrane technology demonstrates a remarkable capability to effectively reduce pollutants, enhance flux stability, and improve scalability compared to traditional membrane methods and commercial photocatalysts.

Notably, the pollution reduction achieved by PEC systems is 3–10 times greater than that of conventional approaches, resulting in significant cost savings. These benefits position PEC as a transformative technology in energy–water nexus applications.

Carbon-based materials are frequently utilized as conductive substrates to support photocatalytic materials. Huang et al. [28] revealed that graphene could enhance the conductivity of TiO_2_/graphene composites by improving interfacial charge transfer via a C-Ti bond to enhance photocatalytic activity. Furthermore, adding carbon fiber results in a threefold increase in the electrical conductivity of CoSb_3_ thermoelectric skutterudite [29]. CNTs have attracted increasing attention among all carbon materials due to their notable enhancement of catalyst conductivity. Gao et al. [30] have demonstrated that even in tiny quantities of 1%, the inclusion of CNTs increased conductivity noticeably. Liu et al. [31] found that a 30% addition of CNTs showed improved conductivity and the best OER performance, with an over-potential of only 240 mV in a KOH solution. In addition, with CNT involvement, more exposed sorption sites were obtained [32], facilitating the catalytic process [33].

TiO_2_ is recognized as the most popular photocatalyst and is extensively used in membrane fabrication due to its remarkable photocatalytic properties, stable structure, and low toxicity. Dense heterojunctions have been formed in the Ti-O-CNTs structure by combining TiO_2_ and CNTs [34]. Shafei et al. [35] proved that the band gap energy decreased from 3.2 to 3.05 eV with TiO_2_ composited with CNTs, significantly enhancing MB removal efficiency. Bai et al. [24] developed a CNT/ZnO/TiO_2_ nanohybrid with a “cob-web”-like structure that presented high mechanical strength and photocatalytic capability. CNTs were used as conductive channels and provided a large surface area for catalyst load. Moreover, the combination mode between TiO_2_ and CNTs was essential for performance improvement.

In the present study, a novel PES/CNTs@TiO_2_ membrane was fabricated. The catalytic layer was designed using CNTs@TiO_2_, while PES was the substrate. The PES/CNTs@TiO_2_ membrane combined photo-electrocatalysis and filtration process. The membrane’s hydrophilicity, water permeability, rejection, and photo-electrocatalysis degradation performance were assessed in a lab-scale filtering mode. The findings of this study offer a new method for effectively removing organic pollutants from water, aiding in the advancement of sustainable water treatment technologies.

## 2. Materials and Methods

### 2.1. Materials

Carbon nanotubes (CNTs) (>95%) were offered by Sigma-Aldrich Co. Ltd. (Shanghai, China). Nitric acid, tetra butyl titanate, glycerol used for the fabrication of CNTs@TiO_2_, Na_2_SO_4_, bovine serum albumin (BSA), methyl orange (MO), methyl blue (MB), sulfamethoxazole (SMX), and florfenicol (FF) used to evaluate the separation and photo-electrocatalysis degradation performance were obtained from Sinopharm Chemical Reagent Co. Ltd. (Shanghai, China). The membrane used in this work was a PES membrane (pore size 0.1 µm with a diameter of 47 mm) purchased from Delvstlab Technology Co., Ltd. (Zhejiang, China). All reagents used above were analytical grade. Methanol (HPLC grade) was purchased from Macklin Biochemical Co., Ltd. (Shanghai, China) for analysis on liquid chromatographs.

### 2.2. Synthesis of CNTs@TiO_2_

Nanotube polydispersity is an essential barrier for applications. Studies have shown that the acidification of carbon nanotubes is efficacious in improving dispersion because the acidification process leads to -OH and -COOH functional groups’ attachment to the CNT surface [36]. Carbon nanotubes were immersed in a 10 M nitric acid solution. The suspension was heated to 180 °C and stirred magnetically in a round-bottom flask with a condenser for 6 h. After cooling, the suspension was washed with distilled water until neutral, and the powder was dried overnight at 100 °C.

Acidified carbon nanotubes with different doses were ultrasonically dispersed in 50 mL ethanol for 1 h. Then, the dispersion, 1 mL tetra butyl titanate, and 10 mL glycerol were added into a stainless steel reactor with PTFE lining and stirred for 20 min. The reactor would be heated at 180 °C for 15 h. The generated powder was washed with ethanol several times and dried overnight in an oven at 60 °C. Then, it was annealed in a muffle furnace at 450 °C for 2 h. The powder collected was CNTs@TiO_2_, used in the subsequent preparation of catalytic membranes. The leaf morphology of CNTs@TiO_2_ synthesized through this method can effectively increase photocatalytic efficiency; additionally, the impact of calcining temperature on photocatalysis can be disregarded [37].

### 2.3. Fabrication of the CNTs@TiO_2_ Membrane and Construction of the Photo-Electrocatalytic System

Different doses of CNTs@TiO_2_ nanocomposites were dispersed in pure water for three hours and then filtered onto a PES membrane by vacuum. The final membranes were dried naturally and used for subsequent characterization and performance evaluation experiments.

**Figure 1:**
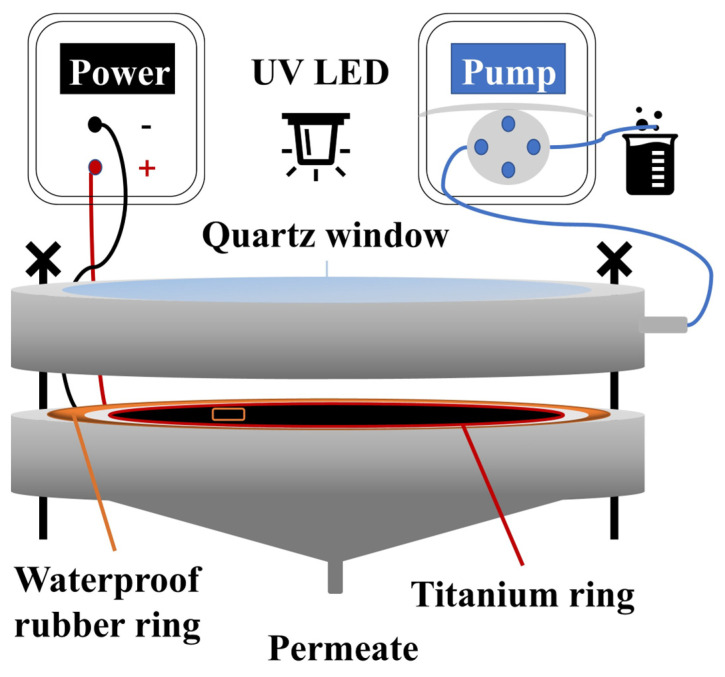
Schematic diagram of photo-electrocatalytic membrane reactor.

A UV LED light was selected as the experimental light source at a peak wavelength of 365 nm. The light transmission window of the membrane reactor was 1 mm thick quartz glass; the light transmittance was above 94%. The central part of the membrane reactor was designed and processed with polymethyl methacrylate. The membrane support layer was a high borosilicate glass filter plate with a thickness of 5 mm and a pore diameter of 16–30 µm. The reactor filtrate drive device was a peristaltic pump purchased from Kamoer Fluid Tech Co., Ltd. (Shanghai, China). The cathode of the photo-electrocatalysis filtration system was a stainless steel mesh placed on the supporting layer, and the anode was a PES/CNTs@TiO_2_ membrane. The cathode and anode were stacked and separated by a PES membrane. The schematic diagram of the photo-electrocatalytic filtration device is shown in Figure 1.

### 2.4. Characterization

The crystal characteristics of CNTs@TiO_2_ were investigated using an X-ray diffractometer (XRD, model XD-3, Beijing Beifen-Ruili Analytical Instrument Co., Ltd., Beijing, China). Surface morphologies of CNTs@TiO_2_ and surface and cross-section morphologies of the catalytic membrane were observed by a Hitach S-4800 scanning electron microscopy (SEM, Hitachi High-Technologies Corporation, Chiyoda-ku, Tokyo, Japan). Energy Dispersive Spectroscopy (EDS) was employed to analyze the types and content of elements in the micro-regional components on the membrane surfaces. A FEI TF20 transmission electron microscope ((TEM, Thermo Fisher Scientific Inc., Hillsboro, OR, USA) was utilized to obtain the microstructure of CNTs@TiO_2_. The surface elemental compositions were analyzed through X-ray photoelectron spectroscopy (XPS, Thermo Fisher Scientific, Chaska, MN, USA). The intensity of light absorbed by TiO_2_ (P25) and CNTs@TiO_2_ with various CNT percentages was measured with a UV-visible spectrophotometer (UV-Vis, Shimadzu Corporation, Kyoto, Japan), performing full spectrum scanning within the wavelength range of 200–800 nm. N_2_ adsorption–desorption isotherms were obtained on an ASAP 2460 instrument (Micromeritics Instrument Corporation, Norcross, GA, USA). The specific surface was obtained using the Brunauer–Emmett–Teller (BET) model, and the mesopore and micropore size distributions were calculated using the Barrett–Joyner–Halenda (BJH) method. The roughness of the PES/CNTs@TiO_2_ membrane was measured using atomic force microscopy (AFM, Bruker Corporation, Billerica, MA, USA).

### 2.5. Analytic Methods

The concentrations of methyl orange (MO), methylene blue (MB), and bovine serum albumin (BSA) were determined using UV-visible spectrophotometry (Puxi Instrument Co., Ltd., Shanghai, China) at wavelengths of 463 nm, 665 nm, and 280 nm, respectively. The concentrations of sulfamethoxazole (SMX) and florfenicol (FF) were determined using a 1290 high-performance liquid chromatography system (Agilent Technologies, Inc., Santa Clara, California, USA) equipped with a C18 column (5 µm, 4.6 × 250 mm). Details about the analytical methods used are provided in Table 2.

### 2.6. Membrane Performance Test

The dynamic contact angles of the PES/CNTs@TiO_2_ membrane were tested using an automatic contact angle meter (Kino Scientific, Boston, MA, USA) to express the hydrophilicity. Pure water flux experiments were conducted for the as-prepared membrane coupons under a direct flow and constant pressure dead-end filtration mode while directly measuring the permeated water flux. Pure water in the storage tank (MilliporeSigma, Billerica, MA, USA) was pressurized by N_2_ at 1 bar. Pure water flux was measured over time by an electronic balance (Sartorius AG, Göttingen, Germany).

(1) $$PWF=\frac{M}{\rho \times A\times T}$$

whereby M is the accumulated permeate water weight, ρ is the density of water, A is the test area of the membrane surface, and T is the test time.

Membrane rejection properties were evaluated by filtering two model contaminants, MO (5 mg·L^−1^, M_w_ = 327.3 Da) and BSA (5 mg·L^−1^, M_w_ = ∼66.5 kDa), as carried out by others. The rejection rate R equals:

(2) $$R=\left(1-\frac{C}{C_{0}}\right)\times 100\%$$

C is the concentration in the permeate, and C_0_ is the initial concentration of feedwater.

CNTs@TiO_2_ was deposited onto a PES membrane substrate, and AFM was employed for direct observation of the surface roughness. The PES/CNTs@TiO_2_ membrane was cut into small pieces measuring 2 cm × 2 cm and placed directly on the sample test bench. Using NanoScope Analysis software (Version 3.00), a scanning area of 1 μm × 1 μm was selected for both spectral analysis and measurement of the membrane roughness. To quantify the roughness, three parameters were evaluated: average roughness (R_a_), which represents the geometric average deviation of the height of the scanning area relative to the datum plane; root mean square roughness (R_q_), which refers to the root mean square value of the height relative to the datum plane; and R_z_, which measures the vertical distance between the highest peak and the lowest valley within the scanning area. These parameters collectively provide a comprehensive understanding of the membrane’s surface roughness.

Equation (3) studied the membrane’s flux recovery rate (FRR). The membrane’s pure water flux was P_1_ (L·m^−2^·h^−1^·bar^−1^). The same membrane was then studied with a 5 mg·L^−1^ MO solution as the target pollutant. After 30 min of filtration, the pure water flux was measured again and denoted as P_2_ (L·m^−2^·h^−1^·bar^−1^).

(3) $$FRR=\left(\frac{P_{2}}{P_{1}}\right)\times 100\%$$

The electrochemical experiments were conducted at room temperature in a cell with a standard three-electrode system using an electrochemical analyzer (CH Instruments, Shanghai, China). Additionally, the three-electrode system comprised a saturated Ag/AgCl reference electrode, a Pt foil counter electrode, and a working electrode (PES/CNTs@TiO_2_ membrane with an active area of 2 cm^2^). The IMP-AC measurements yielded results for electrochemical impedance spectroscopy (EIS). The scanning parameter was set as 1 MHz to 0.1 Hz. A 0.1 M Na_2_SO_4_ solution with 25% methanol was employed as the hole scavenger and the electrolyte. Photocurrents were measured with an LED UV lamp with a peak wavelength of 365 nm.

### 2.7. Capability of the Membrane PECM Process

The photo-electrocatalytic reactivity of the PES/CNTs@TiO_2_ membrane was evaluated by the degradation of several chemicals in flow-through mode. A UV LED light with a peak wavelength of 365 nm and an applied voltage of 0.8 V was utilized for the flow-through test. The target chemicals were filtered under a peristaltic pump pressure. Constant concentration monitoring in the outflow tank was conducted, and the total flux/time was documented. The organic compounds degradation kinetics for different systems were determined using a pseudo-first-order model as described in Equation (4):

(4) $$\ln\frac{C_{t}}{C_{0}}=-k_{app}\times t$$

where K_app_ is the apparent pseudo-first-order rate constant, and C_0_ and C_t_ are the organic compound concentrations at the initial time and following reaction for time t, respectively.

## 3. Results

### 3.1. Morphology and Structural Characterization of CNTs@TiO_2_

**Figure 2:**
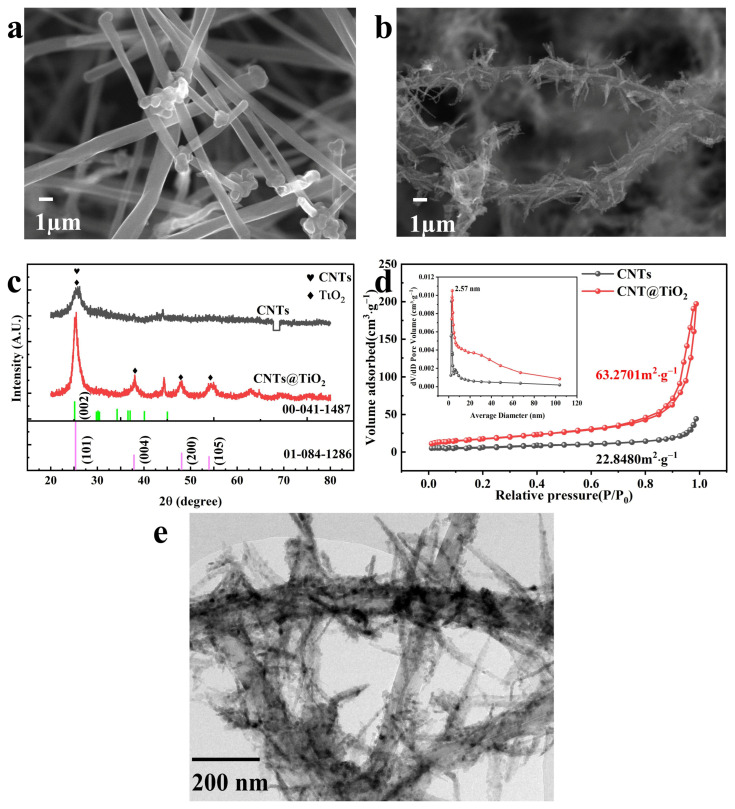
(**a**,**b**) SEM images of CNTs and CNTs@TiO_2_; (**c**) XRD patterns of CNTs and CNTs@TiO_2_; (**d**) N_2_ adsorption-desorption isotherms, BET surface area, and pore size distribution of samples; (**e**) TEM image of CNTs@TiO_2_.

The CNTs@TiO_2_ composite was fabricated using a hydrothermal method, and its morphology was characterized by XRD, SEM, TEM, and BET. A tubular structure with a diameter of 1 µm was observed for pristine CNTs (Figure 2a). A leaf-like bionomic structure was observed with TiO_2_ dispersed on CNT (Figure 2b). This leaf-like structure provides superior adsorption and light harvest capabilities due to the large surface area.

The XRD results showed that the diffraction peak at 2θ = 25.3° was attributed to (002) crystal facets of the CNTs (Figure 2c). For CNTs@TiO_2_, diffraction peaks at 2θ = 25.3°, 37.8°, 48.1°, and 54.0° corresponded to the (101), (004), (200) and (105) planes of TiO_2_ (JCPDF card No. 84-1286), respectively. The diffraction peaks of CNTs@TiO_2_ matched well with those of standard CNTs and TiO_2_ particles, indicating TiO_2_ was deposited on CNTs successfully [37]. The characteristic peak presented at 25.3° of CNTs (002) in CNTs@TiO_2_ was higher than the pristine CNTs, which was mainly due to the overlap peaks of CNTs (002) and anatase (101) [38].

An N_2_ adsorption–desorption isotherm was used to determine the specific surface area and pore volume distribution. Pristine CNTs and CNTs@TiO_2_ samples exhibited a Type IV adsorption isotherm with an N_2_ hysteresis loop in the range (P/P_0_) of 0.6–1.0, demonstrating the typical material mesoporous structure [39]. The specific surface area of CNTs@TiO_2_ was 63.27 m^2^·g^−1^ (Figure 2d), much higher than that of the raw CNTs (22.85 m^2^·g^−1^). The most significant pore size distribution frequency of the CNT@TiO_2_ composite was 2.57 nm, which is not appreciably different from the original CNTs (2.84 nm). This result suggested that the internal structure of CNTs was less affected by the loading of TiO_2_. The pore volumes of CNTs@TiO_2_ (0.1 cm^3^·g^−1^) were more extensive than the pristine CNTs (0.06 cm^3^·g^−1^). The larger specific surface areas and pore volumes provide more space to adsorb N_2_ [40], which provides more adsorption sites and enhanced photocatalytic ability.

The TEM analysis provided detailed insights into the crystalline structure (Figure 2e). We observed that the nanoparticles were well-dispersed and uniform in size. This observation is consistent with the SEM results, where the CNTs@TiO_2_ surface featured well-defined leaf-like structures, which could provide more active sites for catalytic reactions and enhance its overall performance. These findings indicate that our synthesis method effectively controlled the morphology of the CNTs@TiO_2_ particles.

**Figure 3:**
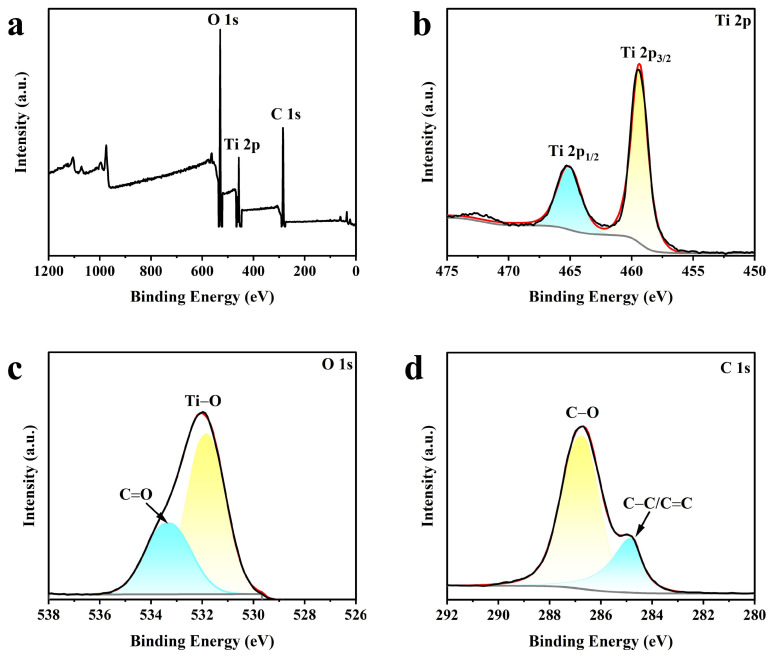
XPS spectra of the CNTs@TiO_2_ powder. (**a**) Survey spectrum, (**b**) Ti 2p, (**c**) O 1s, (**d**) C 1s. The yellow and blue regions represent the fitted peaks, the solid gray line represents the background value, the solid red line represents the fitted signal, and the solid black line represents the measured signal.

XPS measurements were performed to evaluate the surface chemical composition of CNTs@TiO_2_. Figure 3a–d plots the XPS measurement spectra with C 1 s, O 1 s, and Ti 2p. The existence of CNTs@TiO_2_ is confirmed by the XPS spectra’s C 1 s, O 1 s, and Ti 2p peaks. It is evident from the X-ray photoelectron spectra of Ti in Figure 3b that the presence of Ti^4+^ is demonstrated by the two distinctive peaks with binding energies of 459.3 eV and 465.1 eV, which correspond to Ti 2p_3/2_ and Ti 2p_l/2_ of Ti 2p, respectively [41]. From Figure 3c, the peak of binding energy at 531.8 eV in the X-ray photoelectron spectrum is derived from the O^2-^ in TiO_2_ [42]; the peak with a binding energy of 533.3 eV comes from the C=O [43]. In Figure 3d, binding energy peaks at 284.8 eV and 286.7 correspond to the C-C or C=C bind and C-O, respectively [44]. Figure 3a–d shows the spectra of C 1 s, O 1 s, and Ti 2p, which proves that CNT@TiO_2_ was successfully synthesized and had -OH, -COOH function groups.

### 3.2. Photocatalytic Performance of the CNTs@TiO_2_ Composite

**Figure 4:**
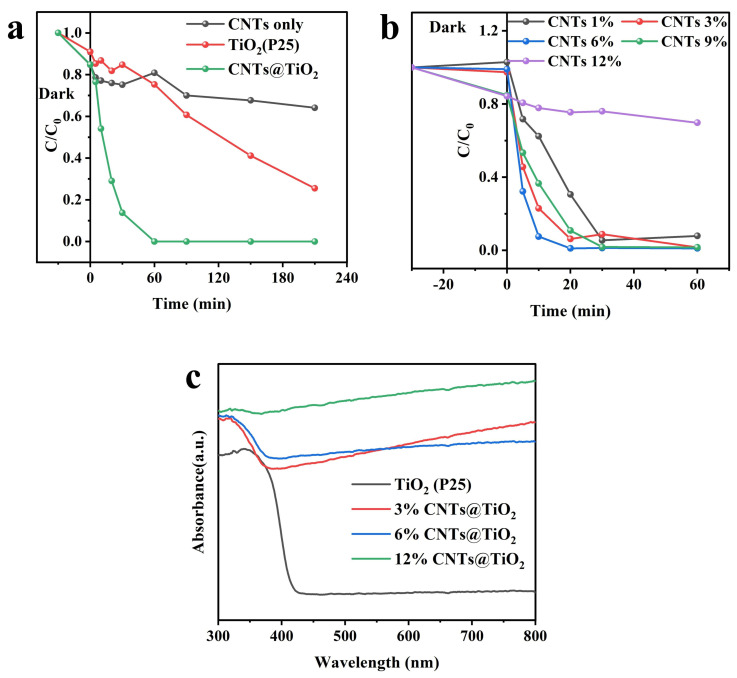
(**a**) Photocatalytic degradation performance of MO with different catalysts; (**b**) photocatalytic degradation performance of MO with CNTs@TiO_2_ of different ratios (C_MO_ = 20 mg·L^−1^, V_MO_ = 50 mL, catalyst dosage = 0.1 g·L^−1^); (**c**) UV adsorption spectra of the TiO_2_ (P25), 3% CNTs@TiO_2_, 6% CNTs@TiO_2_, and 12% CNTs@TiO_2_.

The photocatalytic activity experiments with various catalysts were carried out using a UV LED (Zigu, Zhongshan, China) illumination (365 nm), with 20 mg·L^−1^ MO selected as the target compound. Before the photocatalytic reaction, MO was mixed with the catalysts and stirred in the dark for 30 min to attain absorption equilibrium. The photocatalytic performances of commercial TiO_2_ (P25) and CNTs were compared with those of CNTs@TiO_2_. Approximately 20% of the MO was removed through the adsorption process of CNTs@TiO_2_ and the pristine CNTs. Under illumination conditions, the photocatalytic degradation rate of CNTs@TiO_2_ reached 100% in 60 min (Figure 4a), while only 74.5% and 35.9% were achieved for TiO_2_ (P25) and the CNTs, respectively. The exceptional photocatalytic activity of CNTs@TiO_2_ is primarily attributed to the composite of the CNTs and TiO_2_. According to previous reports, the direct interaction between the CNT surface and the TiO_2_ hybrid structure introduces a new carbon energy level into the TiO_2_ band gap, significantly reducing the band gap energy and enhancing photocatalysis [45].

Additionally, the enhanced photocatalytic ability of CNTs@TiO_2_ is mainly due to the rapid separation of photo-generated electrons and holes. CNTs act as an electron trapper, crucial for the photo-generated electron separation process. Moreover, the biomimetic leaf-like shape enables TiO_2_ to harvest light more efficiently, and the rapid electron transfer rate of CNTs in the composite endows CNT@TiO_2_ with outstanding photocatalytic characteristics.

The influence of the CNT percentage (ranging from 1% to 12%) in the synthesis of CNTs@TiO_2_ composites was investigated, as shown in Figure 4b. A minor increase in the adsorption of MO was observed as the CNT percentage rose when the experiments were conducted in the dark for 30 min. When the CNT percentage reached 12%, the MO removal rate by adsorption was nearly 20%. The photocatalytic degradation rate increased with the increase in the CNT percentage from 1% to 6%. The highest degradation rate was achieved with a CNT percentage of 6%, reaching 100% within 20 min. Nevertheless, when the CNT percentage was further increased to 12%, the MO degradation rate decreased significantly, with only 30% degradation in 60 min. This phenomenon is mainly attributed to the various CNT contents resulting in different structures of CNTs@TiO_2_. With a lower CNT content, the nucleating sites on the CNTs are limited, and the TiO_2_ nanoparticles are prone to blending. According to the literature, as the CNT content increases, the structure of TiO_2_ changes from a broad-leaved to a needle-leaved one [37]. The long and narrow needle-leaved structure formed with a CNT percentage of 6% facilitates light harvesting, and optimal photocatalytic degradation is obtained.

Furthermore, the use of UV-vis spectra is an effective method for analyzing the bandgap width of photocatalytic materials. As shown in Figure 4c, the light absorption intensities of TiO_2_ (P25), 3% CNTs@TiO_2_, 6% CNTs@TiO_2_, and 12% CNTs@TiO_2_ were analyzed through UV-vis full spectrum scanning. The figure shows that TiO_2_ (P25) has an absorption edge at 400nm, corresponding to a bandgap width of about 3.0 eV, which is the intrinsic absorption band edge of TiO_2_ [46]. The light absorption intensity was in the following order: 12% CNTs@TiO_2_ > 6% CNTs@TiO_2_ > 3% CNTs@TiO_2_> TiO_2_ (P25) at 365 nm, which was the same as the experiment condition. Meanwhile, the light absorption intensity increases with a higher percentage of CNTs, due to their exceptional performance in electronic capture and transmission.

The synthesized CNTs@TiO_2_ was loaded on the PES membrane substrate, referring to method 2.3 mentioned previously.

**Figure 5:**
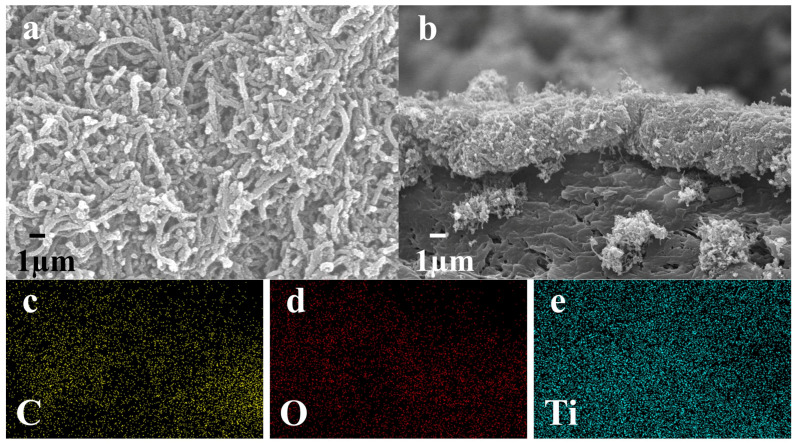
(**a**,**b**) SEM images of the surface and cross section of a PES/CNTs@TiO_2_ membrane; (**c**–**e**) EDS mapping of the three elements (The yellow dots represent the element C, the red dots denote the element O, and the blue dots signify the element Ti).

The surface and cross-section morphology of the PES/CNTs@TiO_2_ membranes were examined by SEM (Figure 5a,b). A loose and porous layer composited by CNTs@TiO_2_ nanoparticles was deposited on the PES membrane surface. With the deposition quantity of 12 mg, the CNTs@TiO_2_ layer had a thickness of approximately 15 µm (Figure 5b). EDS mapping analysis showed that elements C, Ti, and O coexisted with mass percentages of 27.29%, 28.94%, and 43.77%, respectively (Figure 5c–e).

### 3.3. PES/CNTs@TiO_2_ Membrane Performance Analysis

**Figure 6:**
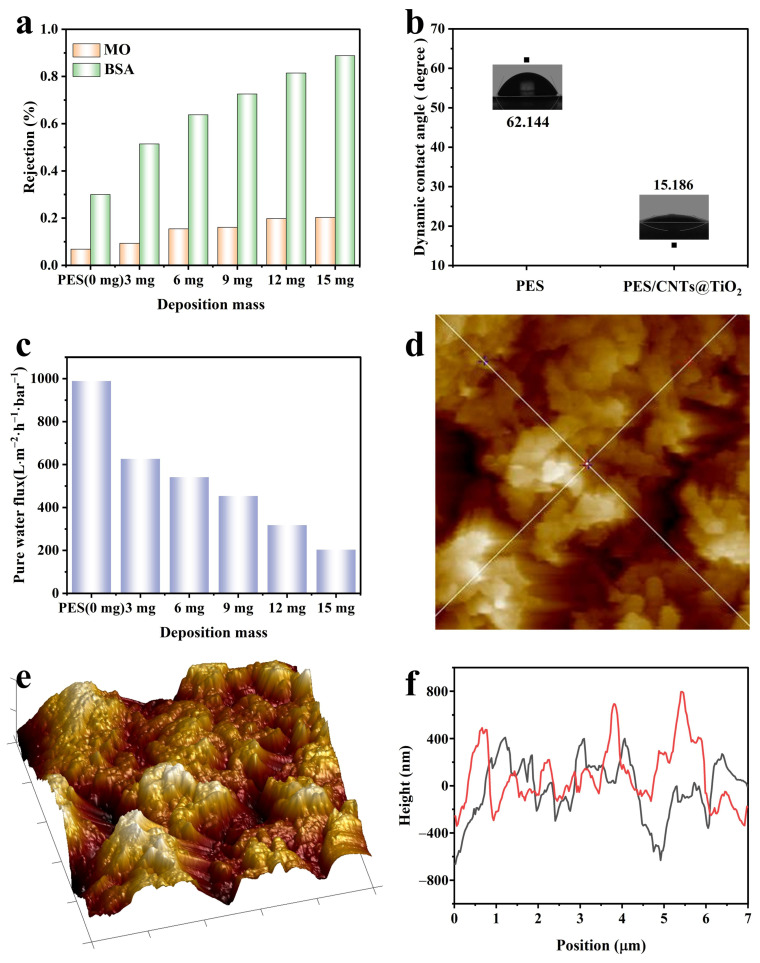
(**a**) Rejection of MO and BSA with different deposited CNTs@TiO_2_ mass; (**b**) dynamic contact angle of the pristine PES membrane and the PES/CNTs@TiO_2_ membrane; (**c**) pure water flux of the PES/CNTs@TiO_2_ membrane with different deposited mass; (**d**,**f**) height variations across the diagonal lines in the membrane surface testing area; (**e**) roughness of the PES/CNTs@TiO_2_ membrane.

The separation performance of PES/CNTs@TiO_2_ membranes was assessed by filtering MO (327 Da) and BSA (~66.5 kDa), which are recognized as typical compounds. The rejection rate of the pristine PES membrane was 6.87% for MO and 30% for BSA (Figure 6a). To eliminate the influence of adsorption, the as-prepared PES/CNT@TiO_2_ membrane was initially adsorbed for 30 min to reach absorption equilibrium in the same solution as the one being tested. The rejection rates of the PES/CNT@TiO_2_ membrane with CNTs@TiO_2_ quantities of 3 mg to 15 mg were 9.33%, 15.44%, 16.13%, 19.83%, and 20.32% for MO, and 51.42%, 63.78%, 72.58%, 81.46%, and 88.79% for BSA. As the deposition amount of CNTs@TiO_2_ increased, the rejection rates for MO and BSA gradually rose, indicating an improvement in the rejection rate of the PES/CNT@TiO_2_ membrane. Furthermore, the different removal efficiencies between MO and BSA suggest that smaller molecules like MO have high diffusion velocities [47], making them more likely to pass through the membrane pores and be detected. Hence, the deposition of CNTs@TiO_2_ has a relatively minor impact on organic compounds with small molecular weights. The rejection rate of BSA increased significantly with the loading amount, indicating that the loading of CNTs@TiO_2_ on the membrane surface impedes the passage of macromolecule substances, thereby substantially impacting their rejection rate.

Surface hydrophilicity can affect membrane permeability and antifouling properties [22], so the dynamic contact angle is employed to characterize the surface hydrophilicity of membranes. As shown in Figure 6b, the dynamic contact angle of the pristine PES membrane was 62.14°. When the deposition of CNTs@TiO_2_ varied from 3 mg to 15 mg, the dynamic contact angles recorded for the membranes were 9.86°, 6.93°, 6.44°, 15.19°, and 9.38°, respectively. The reduction in the dynamic contact angle was primarily attributed to the increase in hydrophilic functional groups with CNT deposition, such as carboxyl and hydroxyl. A thin water layer would be formed on the hydrophilic PES/CNTs@TiO_2_ surface, enhancing the antifouling properties and preventing the adsorption of organic compounds [48].

The thickness and hydrophilicity of CNTs@TiO_2_ significantly impacted the membrane’s pure water flux (PWF). Consistent with prior studies, an inverse relationship between PWF and the loading amounts of catalysts was discovered [49]. In this research, the PWF of the pristine PES membrane was 989.2 L·m^−2^·h^−1^·bar^−1^, and it decreased to 626.4, 541.2, 453.5, 318.2, and 203.8 L·m^−2^·h^−1^·bar^−1^ after loading 3 mg to 15 mg of catalysts on the membrane (Figure 6c). Despite this, the modified membrane’s pure water flux (PWF) remained relatively high compared to earlier reports [22,50]. The high pure water flux could be due to the rigid nonpolar structures of CNTs, which may serve as unique molecular channels for water [51], leading to exceptionally rapid water transport [52].

The roughness of the PES/CNTs@TiO_2_ membrane was observed through AFM, shown in Figure 6e. The obtained R_a_ value was 229 nm, R_q_ was 287 nm, and R_z_ was 2129 nm, indicating that the membrane exhibited a relatively uniform distribution of features. The height variations across the membrane surface were examined (shown in Figure 6d,f), and the results suggest a certain degree of homogeneity. However, some minor local variations were also observed, which could be attributed to the nature of the deposition process or the characteristics of the membrane components.

**Figure 7:**
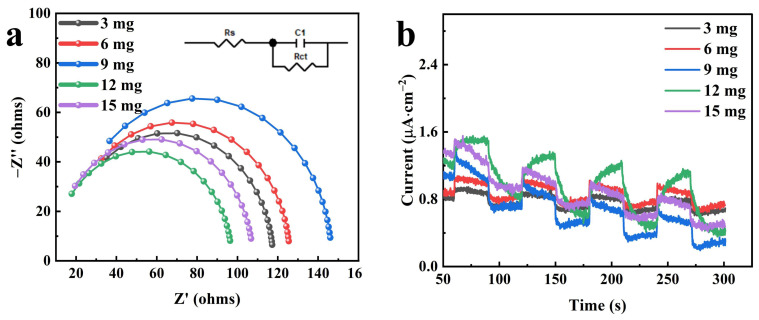
(**a**) Nyquist plots for membranes with different deposited CNTs@TiO_2_ mass; (**b**) photocurrent for membranes with different deposited CNTs@TiO_2_ mass.

Electrochemical impedance spectroscopy (EIS) was employed to investigate the resistance of PES/CNTs@TiO_2_ membranes. In the Nyquist plots, the semi-circular arc is associated with the electrode’s interfacial charge transfer resistance (R_ct_). In contrast, the linear slope in the low-frequency region is related to the diffusion between the electrode and the electrolyte [53]. Figure 7a shows that only a single arc associated with the charge transfer step [54,55] was found in the high-frequency region. The resistance of the PES/CNTs@TiO_2_ membranes was fitted to be 130.4 ohms (3 mg), 111.5 ohms (6 mg), 130.6 ohms (9 mg), 87.99 ohms (12 mg), and 98.1 ohms (15 mg). The lowest resistance value was obtained when the deposition mass of CNTs@TiO_2_ was 12 mg, indicating enhanced electron transfer kinetics [21].

The photocurrent responses for the PES/CNTs@TiO_2_ membranes were investigated as presented in Figure 7b. The photocurrent tested was nearly zero in the dark but emerged rapidly upon exposure to light. Upon turning on the light, the perpendicular shift in the photocurrent indicated the rapid charge transfer speed [56]. The photocurrent peak value increased with the rising loading amounts of CNTs@TiO_2_, demonstrating that adding CNTs@TiO_2_ could enhance the photocatalytic performance by accelerating the photogenerated electron–hole separation at the interface. The most extraordinary photocurrent response was observed with a loading level of 12 mg, which was mainly attributed to the decreased electron–hole recombination rate [57]. As the loading amount of CNTs@TiO_2_ increased, the number of active sites on the membrane surface also increased. However, due to the inhibition of light penetration, the generation of photogenerated electron–hole pairs would be hindered after the loading level reached 15 mg. Along with the extension of the irradiation cycles, a slight attenuation of the photocurrent was noticed.

### 3.4. Performance of the Photo-Electrocatalytical Membranes

**Figure 8:**
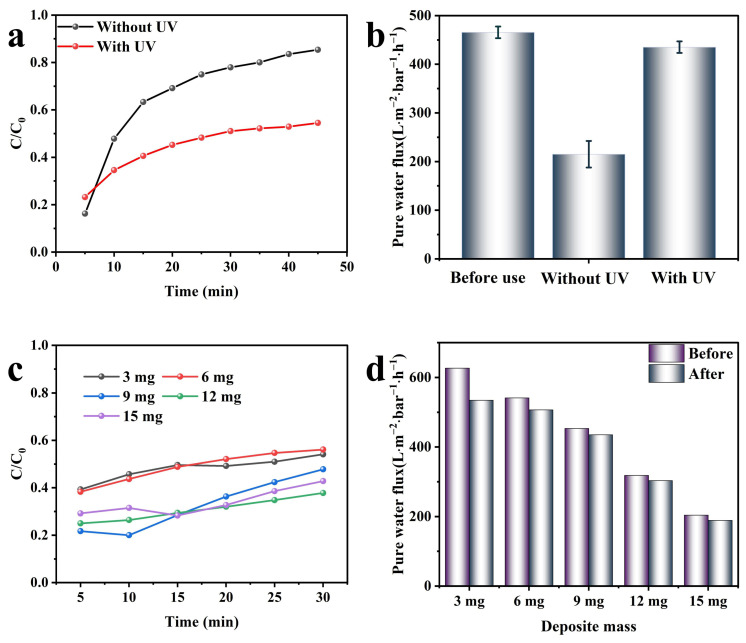
(**a**) MO concentration (outflow tank concentration over inflow concentration C/C_0_) changes with filtered time with/without UV irradiation; (**b**) comparison of PWF with/without UV irradiation; ((**a**,**b**): C_MO_ = 5 mg·L^−1^, V_MO_ = 180 mL); (**c**) comparison of MO concentrations with different deposited CNTs@TiO_2_ mass on the membrane towards photocatalytic process; (**d**) comparison of PWF with different deposited CNTs@TiO_2_ mass on the membrane towards photocatalytic process ((**c**,**d**): C_MO_ = 5 mg·L^−1^, V_MO_ = 50 mL).

The photo-activity of CNTs@TiO_2_ loaded membranes was examined in the flow-through mode. MO was chosen as the target compound, and its concentration in the outflow was evaluated as a function of time with and without UV. The results are presented in Figure 8a. Without UV irradiation, the MO in the outflow reached 16.2% within the first five minutes and increased to 85.4% after a 45 min reaction, with only 14.6% removed. Only the rejection procedure was involved in this process. This result indicated that the removal efficiency of the PES/CNTs@TiO_2_ membrane decreased rapidly during the reaction without UV irradiation.

Nevertheless, the evolution of MO concentration under UV irradiation exhibited a similar trend at lower concentration levels, which can be attributed to the simultaneous photodegradation of MO. After 50 min, the MO in the outflow only slightly increased to 54.5%. The MO removal efficiency was enhanced by approximately 31% through UV illumination at the end of the 180 mL filtrate. Thus, it can be inferred that UV irradiation improves the efficiency of the modified membrane filtration process [58].

Furthermore, the modified membranes’ pure water flux (PWF) was measured before and after a single round of filtration. The flux decreased significantly from 465.5 ± 12 L·m^−2^·h^−1^·bar^−1^ to 215.0 ± 27.3 L·m^−2^·h^−1^·bar^−1^ after the filtration of MO without UV irradiation. Nevertheless, the PWF remained as high as 435.1 ± 11.8 L·m^−2^·h^−1^·bar^−1^ after the filtration of MO with UV irradiation, with an attenuation efficiency of only 6.5% (Figure 8b). The attenuation of the PWF might be attributed to the accumulation of MO on the membrane surface. Under UV irradiation, the MO attached to the membrane surface can decompose into small molecules that are easier to pass through the membrane pores, thereby maintaining the stability of the PWF. Without UV irradiation, the MO accumulated on the membrane surface will impede the PWF and lead to flux loss.

The effects of CNT@TiO_2_ loading amounts on the photocatalytic degradation efficiency were examined. The loading amounts of CNT@TiO_2_ were individually increased from 3 mg to 15 mg, with an effective membrane surface area of 15.9 cm^2^. The MO removal efficiency increased with the rising deposition amount of CNTs@TiO_2_, and the highest photocatalytic degradation efficiency was achieved at a deposition of 12 mg, as depicted in Figure 8c. In line with the previous conclusion drawn in Chapter 3.3, when the CNTs@TiO_2_ deposition amounts reached 12 mg, the modified membranes obtained the lowest resistance, the greatest photocurrent response, and excellent photocatalytic degradation efficiency.

The PWF of the modified membranes with various CNTs@TiO_2_ deposition amounts was analyzed as presented in Figure 8d. A decreasing trend of PWF was noticed with the increase in CNTs@TiO_2_ deposition amounts. As the CNTs@TiO_2_ deposition amounts rose from 3 mg to 15 mg, the corresponding PWF decreased from 626.4 L·m^−2^·h^−1^·bar^−1^ to 203.8 L·m^−2^·h^−1^·bar^−1^. Nevertheless, the difference in permeate water flux (PWF) before and after photocatalytic filtration decreased as the deposition amount increased. The membrane with a loading amount of 3 mg exhibited the highest PWF loss at 14.7%, while the lowest loss of 4.0% was observed with a loading amount of 9 mg. These results suggest that the loss of PWF was primarily due to the accumulation of MO and its intermediates on the membrane surface. The amount of CNTs@TiO_2_ deposited is related to the reactive oxygen species (ROS) generated during the photocatalytic process. This, in turn, affects the efficiency of MO degradation [47], resulting in a variation in pollutant accumulation on the membrane, corresponding to the loss of PWF.

Since the photocatalytic performance is limited by the recombination of photogenerated electrons and holes [25], the application of bias voltage has been recognized as an effective approach to inhibiting the recombination of photogenerated electrons and holes and improving photocatalytic performance. Electrocatalysis, photocatalysis, and photo-electrocatalysis experiments have been carried out to determine the role of bias voltage.

**Figure 9:**
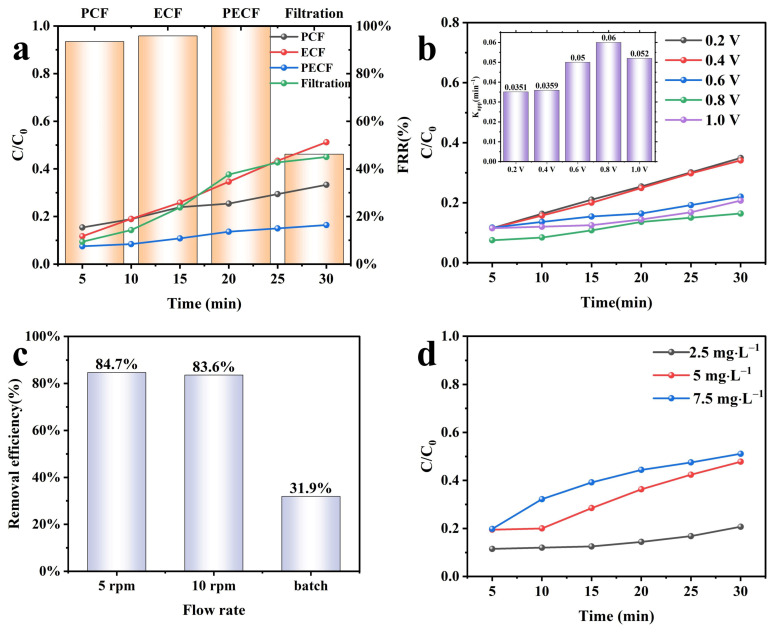
(**a**) MO degradation performance and flux recovery rate under different conditions (C_MO_ = 2.5 mg·L^−1^, V_MO_ = 50 mL, applied voltage = 0.8 V); (**b**) MO degradation performance under different applied voltages; (**c**) MO degradation performance under different flow rates; (**d**) MO degradation performance under different initial concentrations of MO.

In a system where only filtering is applied, the removal rate of MO is 55%. In the electrocatalysis system, the PES/CNTs@TiO_2_ membrane was used as the anode, while the stainless steel mesh was employed as the cathode, and the concentration of MO in the outflow remained at 51% after a 30 min reaction (Figure 9a). In the photocatalysis system, MO remained 33% in the outflow tank after 30 min. The photocatalytic filtration process was superior to the electrocatalytic filtration for MO degradation. In the photo-electrocatalysis system, when a voltage of 0.8 V was applied, the ratio of MO in the effluent dropped even lower to 16%. The best performance was obtained in the photo-electrocatalytic filtration process, indicating an enhanced photocatalytic effect from the applied voltage. Figure 9a also presents the flux recovery rates for four systems: the individual filtration process at 46.19%, photocatalytic filtration at 93.47%, electrocatalytic filtration at 95.86%, and the photocatalytic filtration process at 99.81%. The photo-electrocatalytic filtration process achieved the highest flux recovery rate. This can be attributed to the ROS generated during the process, which degrade the accumulated MO on the membrane surface into smaller molecules. This degradation prevents membrane pore blockage and helps maintain stable flux.

The effects of the applied voltage on the degradation efficiency of MO photo-electrocatalysis were analyzed, as presented in Figure 9b. The removal efficiency of MO was enhanced from 65.1% to 83.6% as the applied voltage increased from 0.2 V to 0.8 V. Nevertheless, when the applied voltage was further raised to 1.0 V, the removal efficiency decreased to 79.3%. The decrease in the MO removal efficiency might be attributed to the side reaction of hydrogen evolution at a voltage of 1.0 V [59]. The literature indicates that the reallocation of the space charge layer and the Helmholtz layer could be the reason for the decreased response rate K_app_. As a consequence of this reallocation, there would be fewer photogenerated h^+^-e^-^ pairs [60]. Hence, if the applied voltage were higher than 0.8 V, the MO degradation rate (K_app_) would decrease rather than increase. Consequently, an applied voltage of 0.8 V resulted in the best removal efficiency, reaching 83.6% within 30 min.

The flow rates of filtrate on MO removal efficiency in the photo-electrocatalysis system were also examined under neutral conditions with an applied potential of 0.8 V (Figure 9c). In comparison to the batch mode, where the removal efficiency of MO was 68.1%, the removal efficiency was higher in the flow-through mode. It was 84.7% at low peristaltic pump speeds (v = 5 rpm) and 83.6% at high speeds (v = 10 rpm). In the flow-through mode, the mass transfer efficiency can be accelerated [61], which is beneficial for the reaction between MO and ROS formed in the system. On the contrary, in the batch mode, the concentration gradient-regulated diffusion mass transfer might block some active sites on the modified membranes, thereby reducing the photo-electrocatalytic degradation performance. The advantage of pollutant removal through photo-electrocatalytic degradation in the flow-through mode is demonstrated by the evidence that the flow of pollutants through the pores leads to an improved transfer to the active sites [62]. Based on the current experimental setup results, the peristaltic pump speed (5 rpm or 10 rpm) in the flow-through mode does not significantly change the MO degradation efficiency.

Then, the influence of different initial concentrations of MO on its removal efficiency was investigated. As shown in Figure 9d, the removal efficiency within 30 min was 79.3%, 52.2%, and 48.9%, with initial MO concentrations of 2.5 mg·L^−1^, 5.0 mg·L^−1^, and 7.5 mg·L^−1^, respectively. Sopajaree et al. [63] have developed a model that predicts that the reaction rate should increase with a decrease in solution concentration in the reservoir, both in the low- and high-substrate concentration regimes of the Langmuir–Hinshelwood kinetics formalism. According to the current research, high pollutant concentrations in the system lead to excessive pollutant adsorption on the catalysts, which hinders the interaction between reactant molecules and photoinduced positive holes or ·OH [64]. Therefore, a lower initial concentration of organic molecules can be employed to ensure the stability of the photo-electrocatalysis efficiency.

### 3.5. Application in the Environment

**Figure 10:**
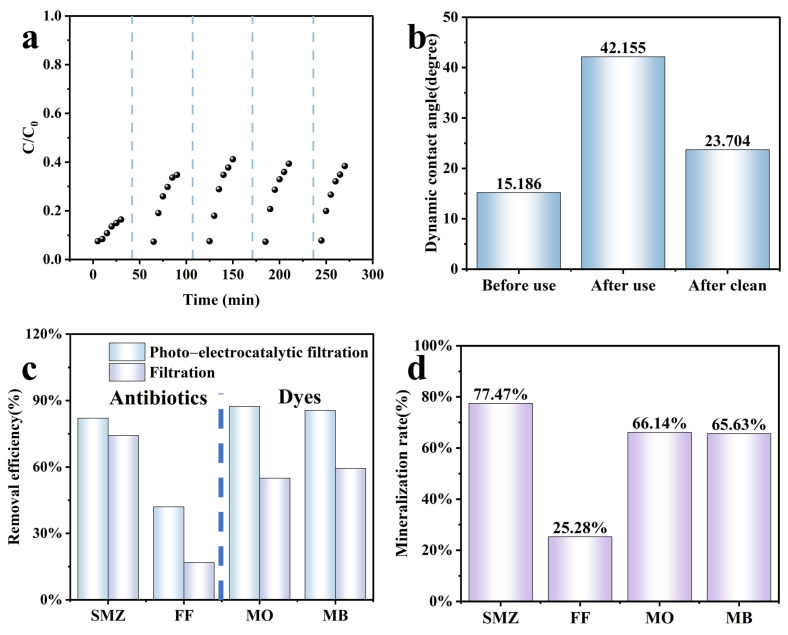
(**a**) Reusability test for photo-electrocatalysis degradation of MO by PES/CNTs@TiO_2_ membrane; (**b**) dynamic contact angle of PES/CNTs@TiO_2_ membrane: pristine, after use and after clean; (**c**) removal efficiency of different pollutants in the photo-electrocatalysis filtration system; (**d**) mineralization rate of four pollutants in the photo-electrocatalysis filtration system. ([SMZ, FF, MO, MB] = 2.5 mg·L^−1^, V = 50 mL, the CNTs@TiO_2_ deposited mass on PES = 12 mg, applied voltage = 0.8 V, solution flow rate = 10 rpm).

Cycle tests for the photo-electrocatalysis degradation system via the PES/CNTs@TiO_2_ membrane (365 nm, 0.8 V) were carried out. After each use, 50 mL of deionized water was employed to clean the modified membranes. As depicted in Figure 10a, with C/C_0_ values of 0.16, 0.35, 0.41, 0.39, and 0.38 in the five test cycles, the corresponding removal efficiency decreased from 83.6% in the first cycle to 61.6% in the fifth cycle. The dependable performance after five cycles indicated the strength and resilience of the PES/CNTs@TiO_2_ membranes against mechanical deterioration and photo corrosion. After each utilization, a rapid water rinse can assist in minimizing membrane fouling, eliminating the organic pollutants accumulated on the surface, and preserving the active surface area for the forthcoming reactions.

The dynamic contact angle of the membranes was observed to increase from 15.19° to 42.16° after five filtering cycles (Figure 10b). The rise in the dynamic contact angle signified a reduction in membrane hydrophilicity. The decreased hydrophilicity might be attributed to the accumulation of organic compounds and their intermediates on the membrane surface, leading to severe membrane fouling. Subsequently, the contact angle reverted to 23.70° after being cleaned with pure water, slightly higher than the initial value. According to the existing literature [65], when water passes through a membrane with a certain roughness on the surface, water and particles penetrate to the bottom of the valleys, and the valleys quickly become “clogged,” resulting in a decrease in roughness. The Wenzel equation shows that the contact angle increases when the surface roughness decreases [66]. Thus, the recovery of the dynamic contact angle of the membrane indicated that the roughness of the membrane surface was restored after cleaning. These findings demonstrated the remarkable stability of the PES/CNTs@TiO_2_ membranes.

The removal efficiency of various organic pollutants in the photo-electrocatalysis system with the PES/CNTs@TiO_2_ membrane was investigated, and the results are presented in Figure 10c. Organic pollutants, such as SMZ (2.5 mg·L^−1^, Mw = 253.278 Da), FF (2.5 mg·L^−1^, Mw = 358.213 Da), MO (2.5 mg·L^−1^, Mw = 327.33 Da), and MB (2.5 mg·L^−1^, Mw = 799.8 Da), were selected as target compounds. The removal efficiencies of the four compounds were 87.4% (MO), 85.6% (MB), 82.1% (SMZ), and 42.0% (FF), respectively. All pollutants were rapidly eliminated within the retention period, except FF. This outcome might be ascribed to the strong C-F bonds with a dissociation energy of 105.4 kcal·mol^−1^, which are difficult to break [67]. Furthermore, Zhang et al. [68] proposed that membrane separation, adsorption, and photocatalysis operate as a synergistic system with a positive interaction among the three processes. In this study, the membrane filtration efficiency of FF was only 16.81%, lower than that of other pollutants tested, resulting in a lower removal efficiency in the photo-electrocatalysis filtration system.

In the PES/CNTs@TiO_2_ photo-electrocatalysis system, oxidation by ROS and filtration jointly contribute to the removal rate of organic pollutants. Therefore, the mineralization rate was also evaluated to investigate the efficiency of organic pollution degradation. The removal rate of total organic carbon (TOC) indicates the mineralization rate. As depicted in Figure 10d, SMZ, FF, MO, and MB mineralization rates were 77.47%, 25.28%, 66.14%, and 65.63%, respectively. Excepting FF, the other three organic compounds can be mineralized by more than 60%. Nevertheless, the tested PES/CNTs@TiO_2_ photo-electrocatalysis system remains a dependable integrated advanced oxidation process with the potential for widespread application.

A comparative analysis of various studies on TiO_2_, polymeric nanocomposite, and ZnO membranes, the findings in Table 3 highlight the performance differences among these membranes, particularly the PES/CNTs@TiO_2_. This novel membrane demonstrates lower rejection rates of small molecules, reducing energy consumption during operation. Notably, its TOC removal efficiency ranges from 25.28% to 77.47%, outperforming TiO_2_ membranes (56% TOC removal) [23] and similar to the efficacy of ZnO membranes [27]. The PES/CNTs@TiO_2_ membrane also shows significant advantages in permeate flux and high flux recovery rates, indicating strong potential for long-term wastewater treatment applications. These results suggest that the PES/CNTs@TiO_2_ membrane is an effective and sustainable option for advanced water treatment technologies.

### 3.6. Degradation Mechanism

**Figure 11:**
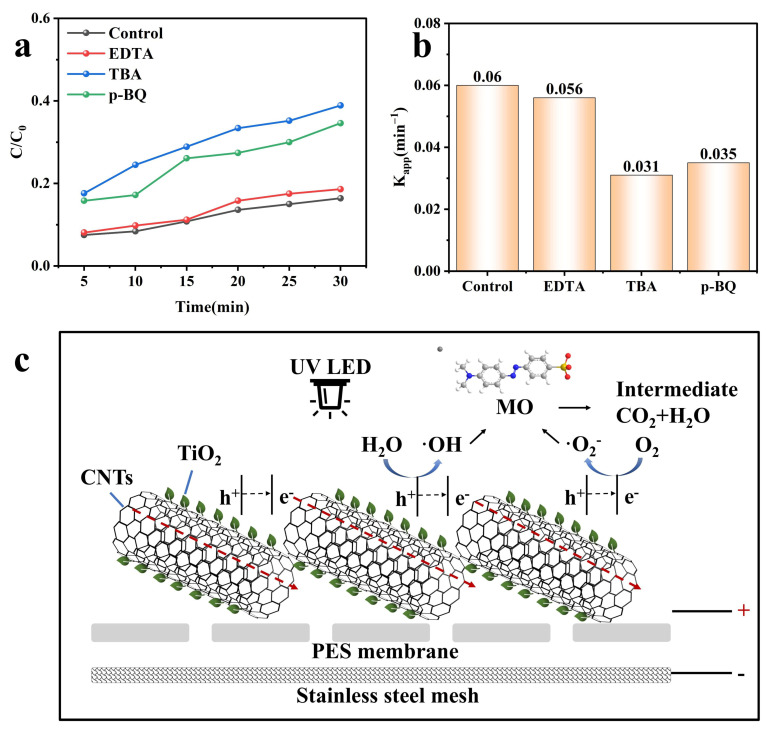
Degradation mechanism: (**a**) PEC degradation of MO in PES/CNTs@TiO_2_ photo-electrocatalysis filtration system with different kinds of scavengers (0.1 mmol⋅L^−1^); (**b**) reaction rate constant k_app_ values (match Figure 10a) with different scavengers; (**c**) schematic diagram of the degradation mechanism. The red dashed line represents the directional migration of photo-generated electrons under an external bias voltage.

To investigate the primary active species and the potential degradation mechanisms of the photo-electrocatalysis filtration system, a series of experiments were conducted to capture active species using various scavengers (0.1 mM each), including EDTA for h^+^, TBA for ·OH, and p-BQ for ·O_2_^−^ [72,73]. As presented in Figure 11a, the removal efficiency of MO was 81.4% (EDTA), 61.1% (TBA), and 65.4% (p-BQ) after 30 min of degradation. Furthermore, the corresponding K_app_ values were 0.056, 0.031, and 0.035 min^−1^ respectively, as shown in Figure 11b. Based on the results, adding EDTA had no noticeable effect on the MO removal efficiency, indicating that h^+^ is not the main active species. Thus, ·OH and ·O_2_^−^ are primarily accountable for the degradation of MO in the PES/CNTs@TiO_2_ photo-electrocatalysis filtration system. The photoelectrons excited by UV irradiation transfer to the conduction band, and subsequently they are utilized to reduce O_2_ and generate ·O_2_^−^. The mechanism of the photo-electrocatalytic filtration system is shown in Figure 11c. The reaction mechanism of MO can be described as follows:

CNTs@TiO_2_ +UV_365_ → e^−^ + h^+^ (5)

O_2_ + e^−^ → ⋅O_2_^−^ (6)

H_2_O + h^+^ → H^+^ + ⋅OH (7)

⋅O_2_^−^ + MO → Intermediate + CO_2_ + H_2_O (8)

⋅OH + MO → Intermediate + CO_2_ + H_2_O (9)

## 4. Conclusions

In this work, a leaf-like bionomic CNTs/TiO_2_ catalyst was successfully synthesized using a hydrothermal process. It was then loaded onto the membrane surface by vacuum filtration to allow for simultaneous photo-electrocatalytic reaction and filtering. Photocatalysis allows for lower effluent concentration and reduced flux loss in the system. The external voltage could enhance photocatalytic degradation performance by preventing photogenerated electron–hole pairs from recombining. The pure water flux of the membrane deteriorated with use, but it could be recovered with a simple wash process. As a result, photocatalyst loading could be used to decrease membrane fouling and increase membrane lifetime. For this reason, constructing a photo-electrocatalytic filtration system could be a viable method for the water treatment industry. However, further research is still needed to increase the efficiency of the separation and degradation of refractory organic pollutants, explore its scalability, and evaluate its long-term effectiveness in real-world water treatment systems.

In conclusion, the PES/CNTs@TiO_2_ membrane shows excellent potential in remediating water contaminated with organic pollutants. However, further studies are needed to explore its capabilities and ensure its complete practical application. These future studies should focus on enhancing the persistent stability of photo-electrocatalysis membranes and the scalability of membrane reactors, which will improve water quality and environmental protection.

## Figures and Tables

**Table 1:** The comparisons between the photo-electrocatalysis filtration system and current membrane technologies or commercial photocatalysts.

Strategy	Pollution Reduction (%) [26]	Energy Consumption	Stability [27]	Scalability	Characteristic
Commercial Photocatalysis	33% (MB) 26.5% (TOC) <5% (TOC)	Light source.	50% flux reduction after 10 days.	Constrained by practical applications	High reactivity, difficult to recycle
Membrane Technology	43% (MB) 35.03% (TOC) 18% (TOC)	Energy conservation.	Good stability	High degree of industrialization.	High separation efficiency, easily contaminated
Integration Technology	94% (MB) 70% (TOC) 56% (TOC)	Light source and electrical energy.	39.6% flux reduction after 10 days.	Great potential for expansion.	Separation and synchronous degradation, high initial cost.

**Table 2:** Details about the analytical methods with HPLC.

Compound	Mobile Phase (%)		Flow Rate (mL·min^−1^)	Wavelength (nm)	Temperature (°C)
SMX	0.1% Phosphoric acid (80)	Acetonitrile (20)	1	240	Room temperature
FF	0.02% Triethylamine aqueous solution (25)	Methanol (75)	1	224	Room temperature

**Table 3:** Performance comparison of various composite membranes.

Membrane	Nanoparticle	Pollutant Removal Efficiency	Rejection %	Pure Water Flux (L·m^−2^·h^−1^·bar^−1^)	Flux Recovery	Long-Term Stability	Ref
PES	-	55% (MO)	6.87% (MO) 30% (BSA)	989.2	46.19%	-	This work
PES	CNTs@TiO_2_	87.4% (MO); 66.14% (TOC); 85.6% (MB); 65.63% (TOC); 82.1% (SMZ); 77.47% (TOC); 42.0% (FF); 25.28% (TOC)	19.83% (MO) 81.46% (BSA)	318.2 (12mg)	93.47% (PCF); 99.81% (PECF)	Maintained a removal rate of 60% after five cycles	This work
PSF	rGO/ZnO	>90% (CR, MO, MB)	-	296	90%	Remained above 90% after 5 h	[12]
PES	NH_2_-MIL-125@MIL-88B	-	96.6	58.4	98%	Maintained a 90% rejection after five cycles	[23]
CA	CNT/ZnO/TiO_2_	4 times faster than P25	82%	-	-	-	[24]
PVDF	MoO_3_/ZnO/GO	73.08%(BPA), 73.65% (Gabapentin), 100% (Diclofenac), 95.57% (Caffeine), 53.50% (nonylphenol)	96.07% (BPA)	40	-	Achieved 74.02% regeneration efficiency after 3 cycles	[27]
α-Al_2_O_3_	N-doped TiO_2_	90% (CBZ)	-	-	-	-	[61]
AAO	TiO_2_	>80% (MB) <30% (MB)	-	25 600	-	40% in first 10 h, 30% after 20 h	[62]
Al_2_O_3_	TiO_2_/carbon	56% (TOC)	74.8% (89.4 nm), 95.1% (198.3 nm) and 97.3% (528.4 nm)	314	-	-	[69]
PSF	GO-vanillin	-	84–90%	91	90.32%	-	[70]
PVDF	ZnO	-	35g/L saline solution 99.9%	4.0 kg·m^−2^·h^−1^	-	Minimal flux reduction over 24 h	[71]

## Data Availability

The original contributions presented in this study are included in the article. Further inquiries can be directed to the corresponding authors.
